# Severe Neonatal Anemia Due to Spontaneous Massive Fetomaternal Hemorrhage at Term: An Illustrative Case with Suspected Antenatal Diagnosis and Brief Review of Current Knowledge

**DOI:** 10.3390/medicina57121285

**Published:** 2021-11-23

**Authors:** Nicolae Gică, Radu Botezatu, Mihaela Demetrian, Ana Maria Vayna, Brîndușa Ana Cimpoca-Raptis, Anca Marina Ciobanu, Corina Gica, Gheorghe Peltecu, Anca Maria Panaitescu

**Affiliations:** 1 Carol Davila Department of Obstetrics and Gynecology, University of Medicine and Pharmacy, 020021 Bucharest, Romania; gica.nicolae@umfcd.ro (N.G.); radu.botezatu@umfcd.ro (R.B.); brindusa.cimpoca@yahoo.com (B.A.C.-R.); anca.ciobanu@umfcd.ro (A.M.C.); mat.corina@gmail.com (C.G.); gheorghe.peltecu@umfcd.ro (G.P.); 2 Filantropia Clinical Hospital, 011171 Bucharest, Romania; mdemetrian@yahoo.com (M.D.); anamariavayna@gmail.com (A.M.V.)

**Keywords:** fetomaternal hemorrhage, fetoplacental hemorrhage, fetomaternal bleeding, Kleihauer–Betke test, fetal anemia, cardiotocography, reduced fetal movements

## Abstract

Fetomaternal hemorrhage is defined as transfer of fetal blood into placental circulation and therefore into maternal circulation during pregnancy, and represents an important contributor to intrauterine fetal demise and neonatal death. The condition is rarely diagnosed prenatally because clinical findings are often nonspecific, and it is unpredictable. In this paper we present an illustrative case of massive spontaneous fetomaternal hemorrhage where the diagnosis was highly suspected antenatally based on maternal reported reduced fetal movements, abnormal suggestive cardiotocographic trace, and increased peak systolic velocity in the fetal middle cerebral artery. We discuss obstetrical and neonatal management and review the current knowledge in the literature. Maintaining a high index of suspicion for this condition allows the obstetrician to plan for adequate diagnostic tests, arrange intrauterine treatment or delivery, and prepare the neonatal team.

## 1. Introduction

Spontaneous fetomaternal hemorrhage (FMH) is defined as fetal blood transfer into the maternal circulation without any history of trauma and without clinical or histopathological evidence of placental abruption. The vast majority of spontaneous FMHs are low-volume bleedings with no hemodynamic significance but can cause alloimmunization. The frequency and volume of these types of transfusions increase with advancing gestational age, reaching a peak at birth [1].

There is no universally accepted threshold to establish fetal erythrocytes volume in maternal circulation to define a small versus massive FMH. Although volumes of up to 150 mL have been proposed to define massive FMH [2], the volume of bleeding should be interpreted in relation to total fetoplacental blood volume, which correlates with fetal size and gestational age. The fetoplacental blood volume is approximately 120 mL/kg estimated fetal weight before 32 weeks and 100 mL/kg after 32 weeks [3]. Thus, a 40 mL FMH for a fetus at 33 weeks with an estimated fetal weight of 2000 g represents a bleeding of 20 mL/kg, which consists of 20% of its fetoplacental blood volume.

One limitation of these definitions is that they do not consider the rate of blood loss or chronic bleedings, which are additional but unmeasurable factors that affect fetal outcome.

The incidence of massive fetomaternal transfusion is difficult to establish, as the clinical findings are nonspecific and unpredictable. It has potential unfortunate outcomes such as severe fetal anemia, hydrops, encephalopathy or even stillbirth. It has been documented that when a large number of Rhesus (Rh) negative women were routinely tested for FMH after birth, the frequency of FMH > 80 mL ranged from 1/1146 to 1/1429 births [2,4].

The pathogenesis of massive spontaneous FMH is not fully understood. It is well known that minute bidirectional blood flow between mother and fetus occurs in physiological pregnancies [5]. However, what triggers increased volume of transfusion without any history of trauma is still to be established. Placental studies from pregnancies with Rh-negative mothers and Rh-positive fetuses showed a correlation between fetal cells in maternal circulation and placental lesions such as intervillous thrombosis, Kline’s hemorrhages, placental infarcts, and retroplacental hemorrhages. Placentas with villous edema and nucleated red blood cells in fetal vessels and villous dysmaturity or immaturity were associated with greater volumes of FMH and higher severity of clinical presentation [5,6].

Early detection of FMH is essential. Almost half of the cases of massive FMH are diagnosed only in the early neonatal period or present as fetal death.

## 2. Aim

This article reviews the clinical presentation, diagnosis, management, and prognosis of complicated FMH pregnancies. A clinical case of massive FMH is presented.

**Case presentation**

**Figure 1:**
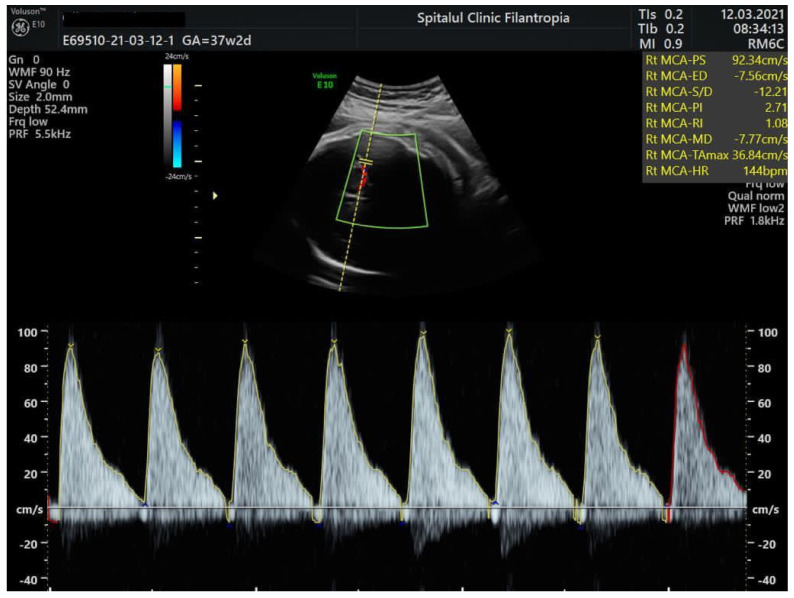
Doppler studies of mean cerebral artery (MCA) in a term baby showing increased peak systolic velocity (PSV) and absent end-diastolic flow.

We present the case of a 32-year-old, para 1, gravida 2 woman with blood type B Rh positive who was admitted to our unit at 37 weeks and 2 days for reduced fetal movements. Until this hospitalization, her pregnancy was uneventful. Prenatal screening tests were unremarkable, and Group B Streptococcus and serologies for congenital infections were negative. On physical examination, there were no other maternal signs or complaints no uterine contractions, and blood pressure and heart rate were normal. Cervix was long and closed with no signs of vaginal bleeding. Ultrasound examination at admission showed an estimated fetal weight of 2900 g, normal amniotic fluid, and normal flow in the umbilical artery, but abnormal Doppler studies in the fetal middle cerebral artery showed (MCA) peak systolic velocity (PSV) of 109.9 cm/s (Figure 1). For the gestational age, this value exceeded 1.5 MoM and was in keeping with severe fetal anemia. Fetal hemoglobin level was estimated based on this measurement at 4.5 g/dL according to the Fetal Medicine Foundation prediction of anemia algorithm. (https://fetalmedicine.org/research/assess/anemia, accessed on 12 March 2021).

**Figure 2:**
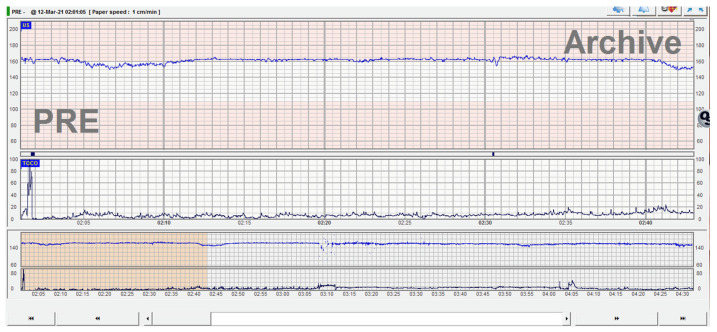
CTG trace with lack of acceleration, absent fetal movements, low variability, and tachycardia.

Fetal cardiac activity on cardiotocography (CTG) recording showed tachycardia, low variability, and chemoreceptor-type decelerations, highlighting the possibility of chronic fetal cerebral hypoxia. There was lack of accelerations and absence of active fetal movements (Figure 2).

**Figure 3:**
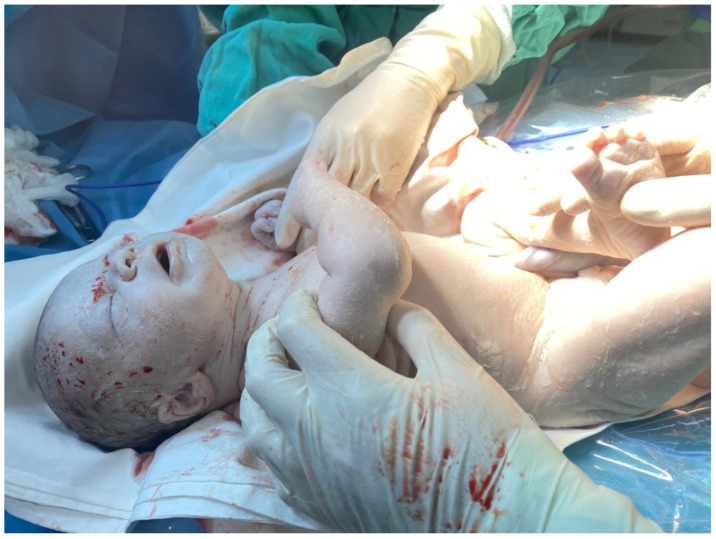
“Ghost-like” appearance of the baby after delivery showing pale skin and lips.

The decision for urgent delivery was made for severe fetal anemia and non-reassuring CTG pattern due to suspected fetal-maternal bleeding; an alive male fetus with a birth weight of 3050 g was delivered. There was no evidence of placental abruption, placenta was pale but otherwise normal, and amniotic fluid was clear. Apgar scores at birth improved from 4 to 6 at 5 min. The newborn required extensive resuscitation in the delivery room, which included positive pressure ventilation and correction of hypovolemia with saline solution. The baby was admitted to the neonatal intensive care unit (NICU). Clinical examination revealed: an extremely pale skin, “ghost-like” (Figure 3) appearance, reduced spontaneous breathing, and respiratory effort, and mechanical ventilation in synchronous intermittent positive pressure mode was required; blood pressure was 41/29 mm Hg (mean arterial pressure 30 mm Hg), oxygen saturation 92%, there was a prolonged capillary recoloration time, tissue perfusion index of <0.5, with no clinical signs of hepatosplenomegaly. There were no peripheral edemas. Correction of the hypovolemia and metabolic acidosis was started. Initially the analysis of arterial blood gases showed metabolic acidosis, a pH of 7.12, PCO2 of 41 mm Hg, base excess of −15. The first hemogram collected from venous blood in the first hour of life showed a hematocrit of 12.8%; hemoglobin was 3.6 g/dL, total leukocytes 53,000/mm^3^, and platelets 178,000/mm^3^. Blood smear analysis later confirmed that most of the leukocytes counted automatically were in fact erythroblasts and an increased number of circulating reticulocytes, suggesting that FMH occurred 1 to 2 days before birth. Blood cultures were negative. A blood sample was taken to establish the etiology of the neonatal anemia. Serology for toxoplasma, rubella, cytomegalovirus congenital infections, and parvovirus B19 had negative results; anti-erythrocyte antibodies were absent, and the Coombs test was negative. Transfusion with erythrocyte concentrate group B Rh-positive was given. Due to the severe anemia and hypovolemic status of the newborn, the transfused volume was 90 mL, calculated according to the formula: the volume of packed red blood cells to be transfused = 1.6 × patient’s weight × (desired hematocrit − patient hematocrit). Despite requiring a major volume for transfusion, because the neonate was at term and the risk of fluid overload and further cardiac decompensation was estimated to be low, we did not use a ‘reversed partial exchange transfusion’.

Posttransfusion hematocrit was 32%. Under treatment, the condition of the newborn was clinically improved, and allowed extubating at approximately 28 h of life and non-invasive ventilatory support (high-humidified flow nasal canula) thereafter.

The echocardiography performed on the first day of life showed a normal heart function and normal heart structures. Transfontanellar ultrasound on the 1st and 5th day of life showed normal brain structures. The baby was discharged after 15 days and had a normal development for age at 6 months postnatal follow-up.

Determination of fetal hemoglobin in maternal blood by the anti-HbF flow cytometry method showed a value of 98.6‰, corresponding to a quantity of 490 mL of fetal blood that was transferred transplacentally to maternal blood, a total volume larger than the blood volume calculated for the newborn by his weight. A summary of the findings in this case is given in Table 1.

## 3. Discussion

Massive FMH is a rare event. Fetal outcome depends on the volume of blood lost and especially if the loss is acute or chronic. In the case presented, no risk factors were identified. The pregnancy progressed to term without pathological elements. The initial and most significant warning sign was the mother’s report of reduced fetal movements. Cardiotocographic evaluation and Doppler studies in the MCA showed very suggestive signs for fetal anemia. Severe anemia (3.6 g/dL) and the cardiovascular status of the newborn (hypovolemia, metabolic acidosis) suggested that the event that led to the massive loss of fetal blood resolved relatively quickly and the fetal compensatory mechanisms functioned. Confirmation of the diagnosis of massive FMH was made by flow cytometry and demonstrated that the lost flow was approximately 490 mL.

FMH can occur at any time during pregnancy. Clinical signs are nonspecific and unpredictable, and sadly, massive acute bleeding can cause fetal death. Hydrops, abnormal fetal heart rate, and decreased fetal movement can be signs of massive but non-lethal or chronic intermittent acute bleeding, causing large cumulative blood loss over time. Reduced or absent fetal movements are the most common symptom of massive FMH. Usually, the mother is asymptomatic, but occasionally symptoms appear and might suggest typical transfusion reaction (fever, chills, nausea) [7].

Clinical examination, nonstress test or CTG and routine ultrasound examination are often insufficient. Reduced fetal movements are nonspecific for fetal anemia [8]. The CTG may present nonspecific deceleration patterns. The sinusoidal model may indicate that fetal anemia should be suspected and treated [9], but this aspect is sometimes correlated with anemia by Rh alloimmunization, where the progress is slow and there is sufficient time for specific changes on CTG to appear. Anther CTG change when fetal anemia may be suspected is the “sawtooth pattern”, a CTG trace that occurs in both fetomaternal hemorrhage and fetal hypotension [10,11]. Other CTG findings include absence of accelerations, recurrent late decelerations, and fetal tachycardia by activating fetal compensatory pathway of adrenergic type—catecholamine secretion in response to hypoxic stress with fetal vascular redistribution. However, if the fetus is compensating sufficiently, the appearance of the CTG trace and the biophysical profile may remain normal.

Peak systolic velocity of the middle cerebral artery (MCA-PSV) in Doppler measurement plays an important role in assessing and monitoring suspected fetal anemia [12]. FMH is associated with MCA-PSV ≥ 1.5 multiple median (MoM), as it correlates with moderate or severe fetal anemia. Doppler evaluation of fetal MCA-PSV is based on the principle that MCA-PSV increases with decreasing fetal hemoglobin levels [13].

Detection of fetal cells in maternal blood is required to confirm FMH. The most widely used test remains the Kleihauer–Betke test. Other methods are flow cytometry and liquid chromatography [14,15,16]. Depending on the obtained result, the fetal blood volume lost into maternal circulation can be estimated according to Mollison’s formula: (percentage of fetal erythrocytes × 1800)/100 × 1.22 = mL of fetal blood [17].

Other clinical scenarios that lead to the diagnosis of massive FMH include:

- Unexplained neonatal anemia. Massive peripartum FMH has been reported in approximately 1 in 9000 births. In severe cases, the newborn may be pale and in circulatory collapse at birth. Laboratory results show an increased production of erythrocyte precursors in fetal blood and an increased number of circulating reticulocytes, suggesting that FMH occurred 1 to 2 days before birth [18].
- Unexpected fetal death. Testing for FMH may be part of the assessment of unexpected fetal death. Up to 15% of fetal deaths are associated with massive FMH [19,20]. The causes are supported by FMH > 20–25 mL/kg fetal weight, and especially > 40 mL/kg fetal weight.
- Nonimmune fetal hydrops (NIFH). NIFH may be discovered by chance during prenatal ultrasound examination performed for standard obstetric care or assessment of reduced fetal movements or abnormal results during routine antepartum pregnancy surveillance: large for gestational age baby or fetal abnormalities associated with hydrops. NIFH evaluation may reveal severe fetal anemia (MCA-PSV ≥ 1.5 MoMs), and subsequent evaluation may determine the cause as massive FMH [21].

Management of massive FMH depends mainly on gestational age at diagnosis, the availability of cordocentesis, which allows fetal blood sampling to diagnose fetal anemia and to perform fetal transfusion, if necessary, and the capacity of the neonatal intensive care unit (NICU). When a 34–36 week pregnancy is complicated with FMH and there are signs of fetal damage, immediate birth is justified. Correction of anemia should be considered before 32 weeks of gestation. After 32 weeks, the risks related to intrauterine transfusion (IUT) should be balanced with the risks of prematurity and/or neonatal transfusion [22]. When FMH occurs before 32 weeks, intrauterine transfusion is a well-established procedure for treating fetal anemia [23], but it is still unclear how to correctly monitor these pregnancies after first IUT or determine the recurrence rate. A retrospective study conducted to investigate the incidence of recurrence after one transfusion found that approximately 71.4% of cases required multiple transfusions. The interval between the first and second transfusion was about 7 days and increased after the second IUT [14].

In the case presented, the suspicion of FMH had to be confirmed by the presence of fetal cells in the maternal bloodstream. Kleihauer–Betke is the most widely used test. The test result quantifies the percentage of fetal cells observed under a microscope. Flow cytometry is an alternative quantitative test. It is based on the fetal cell count (Hb F) linked to monoclonal antibodies, measured by fluorescence intensity. It has better accuracy than the Kleihauer–Betke test but may not be available in all centers.

It is important to note that both tests are unable to address two crucial questions: when did the bleeding occur and whether it was an acute or chronic hemorrhage. This has a major impact on treatment and neonatal outcome as well as on the long-term outcome for the child [24].

## 4. Conclusions

Fetomaternal hemorrhage is a rare condition, but the actual incidence is probably little reported. Some risk factors are well-identified, and in these situations the diagnosis is made immediately. However, in some cases, no risk factors are found, and the clinical findings are not sufficient to make the full diagnosis before birth. Reduced fetal movements, abnormal suggestive cardiotocographic trace, and increased peak systolic velocity in the fetal middle cerebral artery can orientate the obstetrician to this diagnosis. Maintaining a high index of suspicion allows the obstetrician to plan for adequate diagnostic tests, including fetal blood sampling by cordocentesis earlier in gestation, arrange intrauterine treatment (blood transfusion) or delivery, and prepare the neonatal team.

## Figures and Tables

**Table 1:** Summary of the findings in our case of severe fetomaternal hemorrhage.

Antenatal findings	GA 37 wks.; presentation for reduced fetal movements - MCA-PSV—109.9 cm/s (>1.5 MoM; estimated fetal Hb 4.5 g/dL) (Figure 1) - CTG: tachycardia, reduced variability, chemoreceptor type decelerations (Figure 2)
Delivery	Emergency caesarian section Birth weight—3150 g “Ghost-like appearance” baby (Figure 3) Apgar 4 and 6 at 1 and 5 min Resuscitation
Postnatal evaluation	Hb—3.6 g/dL; Ht 12%
Transfusion volume	90 mL
Estimated volume of fetal to maternal hemorrhage	490 mL

GA—gestational age; wks.—weeks; MCA-PSV—middle cerebral artery peak systolic velocity; MoM—multiple of the median; Hb—hemoglobin; CTG cardiotocography; Ht—hematocrit.

## Data Availability

Not applicable.
